# Occult Small Bowel Injury With Delayed Retroperitoneal Abscess Formation After Blunt Trauma: A Case Report

**DOI:** 10.7759/cureus.105446

**Published:** 2026-03-18

**Authors:** Vlasios Gourgiotis, Georgios Stavrou, Sara Upponi, Stavros Gourgiotis

**Affiliations:** 1 Division of Immunology, University of Manchester, Manchester, GBR; 2 Department of Surgery, Addenbrooke's Hospital, Cambridge University Hospitals NHS Foundation Trust, Cambridge, GBR; 3 Department of Radiology, Addenbrooke's Hospital, Cambridge University Hospitals NHS Foundation Trust, Cambridge, GBR

**Keywords:** abdominal trauma, emergency surgery, imaging, laparotomy, pneumo-retroperitoneum

## Abstract

Blunt abdominal trauma can result in bowel injury, but diagnosis is often delayed because early clinical and radiological findings are non-specific. We report a 64-year-old man with pelvic fractures following a road traffic collision who was initially haemodynamically stable, with no definite gastrointestinal injury on computed tomography. Subsequent imaging demonstrated retroperitoneal gas adjacent to a psoas haematoma, without clear evidence of hollow viscus perforation. Despite remaining clinically well, he developed sepsis on day seven post-injury. Repeat imaging revealed progression of retroperitoneal gas, prompting exploratory laparotomy, which revealed a pelvic abscess and non-viable ileum without an identifiable perforation. This case highlights the potential for delayed occult bowel injury following blunt trauma and underscores the importance of sustained clinical vigilance, particularly in patients with complex anatomy or prior abdominal surgery.

## Introduction

Trauma is the leading cause of death in children and adults aged 44 years and under in England and Wales, accounting for approximately 12,500 deaths annually [1]. Blunt abdominal trauma is a common mechanism of injury in high-energy incidents such as road traffic collisions and can lead to hollow viscus injuries that are diagnostically challenging [2].

Bowel and mesenteric injuries occur in approximately 1% of patients sustaining blunt abdominal trauma [3]. Although relatively uncommon, these injuries are associated with significant morbidity and mortality, particularly when diagnosis is delayed [4]. Mortality rates have been reported to increase substantially when operative intervention is delayed beyond 24 hours from injury [5].

Clinical signs are often subtle or absent in the early post-injury period due to altered physiology, concurrent injuries, analgesia, or reduced inflammatory response [6]. Computed tomography (CT) is the imaging modality of choice in haemodynamically stable patients; however, its sensitivity for bowel injury is limited, particularly in the absence of free intraperitoneal air or contrast extravasation [7]. Subtle CT findings such as mesenteric stranding, bowel wall thickening, or isolated free fluid may be overlooked or misattributed to other injuries [8].

Delayed bowel injury may occur secondary to mural contusion, mesenteric vascular compromise, or progressive ischaemic necrosis, leading to perforation or abscess formation days after the initial trauma [9]. Prior abdominal surgery may further obscure both clinical and radiological assessment by altering anatomical planes and limiting the reliability of expected imaging findings [10].

This report describes a markedly delayed retroperitoneal abscess resulting from occult ileal injury, highlighting diagnostic pitfalls, the importance of serial assessment, and structured interpretation of subtle imaging findings.

## Case presentation

A 64-year-old Caucasian male was referred to our Level 1 trauma centre from a District General Hospital (DGH) following a road traffic collision that had occurred six hours before presentation. He sustained significant pelvic trauma. The patient had no significant comorbidities. His past surgical history included anterior resection with loop ileostomy formation and subsequent reversal five years before the current procedure for colonic adenocarcinoma.

At the DGH, the patient was haemodynamically stable with minimal intervention. Primary survey identified right lower quadrant abdominal tenderness and gross pelvic deformity, prompting transfer to a major trauma centre (MTC). Trauma series CT demonstrated haemorrhage adjacent to the right psoas muscle, a right-sided sacral fracture extending to the sacroiliac joint, fractures of the superior and inferior pubic rami, and a displaced right acetabular fracture with pubic symphysis diastasis.

On arrival at our MTC, the patient was tachycardic (101 beats/minute), but otherwise physiologically stable (blood pressure 115/75 mmHg, respiratory rate 18 breaths/minute, oxygen saturation adequate on room air). Abdominal examination revealed a soft abdomen with mild right lower quadrant tenderness, and a pelvic binder was in situ.

**Figure 1:**
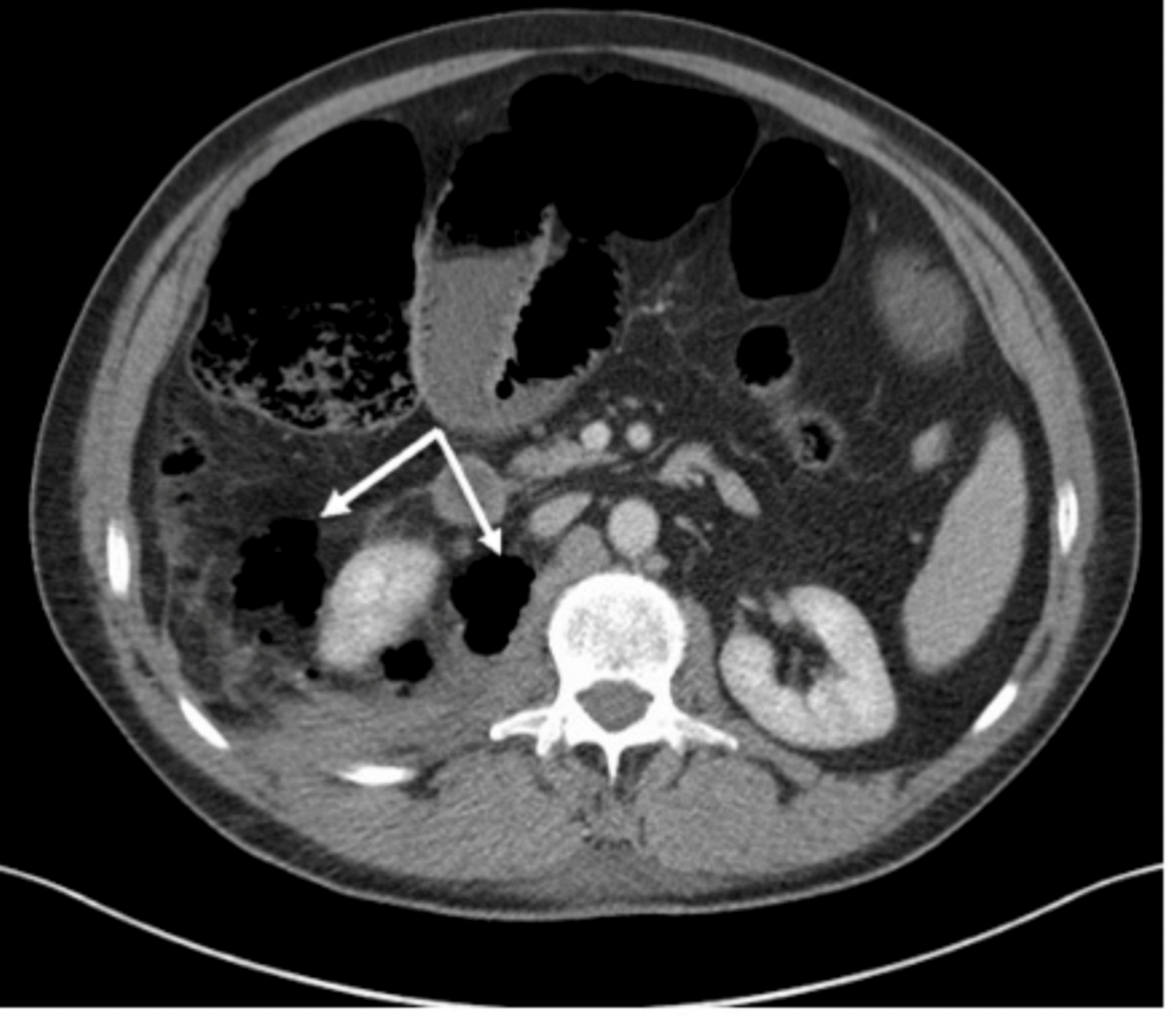
Cross-sectional imaging of the abdomen performed 36 hours after admission in the MTC identified a new large volume of air in the pararenal space, with a few locules of air adjacent to the duodenum and no evidence of duodenal perforation (arrows). MTC, major trauma centre

An interval CT scan with intravenous contrast performed 36 hours after admission revealed a large volume of gas in the right posterior pararenal space adjacent to the stable psoas haematoma, with gas extending retroperitoneally into the pelvis. A small number of gas locules were noted near the duodenum and inferior vena cava, without definite evidence of duodenal injury. Bowel wall thickening was absent, while a mesenteric hematoma was noted. The early psoas collection initially showed no rim enhancement or gas-fluid levels (Figure 1). 

Clinically, the patient’s abdominal pain had resolved. He was tolerating an oral diet, passing flatus, and denied nausea. Abdominal examination remained soft and non-tender. Although consideration was given to bowel rest and parenteral nutrition, the reassuring clinical findings supported continuation of oral intake. Following specialist orthopaedic review, his pelvic injuries were managed conservatively, and he was monitored in a high-dependency unit (HDU).

**Figure 2:**
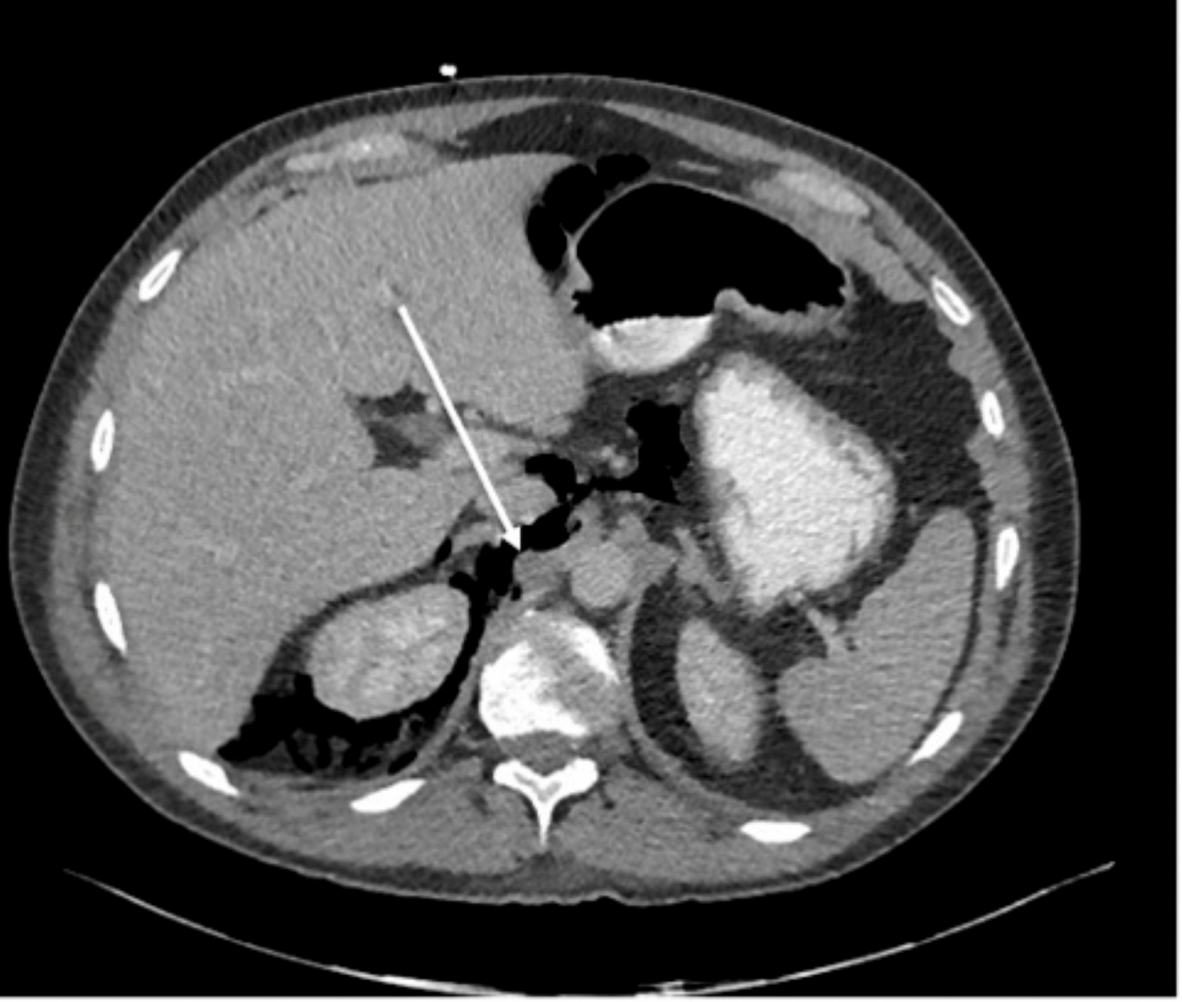
Cross-sectional imaging of the abdomen seven days after trauma demonstrated an interval increase in retroperitoneal free gas, with a new retroperitoneal pelvic collection (arrow).

On day 7 post-admission, the patient developed pyrexia and tachycardia, prompting repeat CT imaging. This demonstrated a new large volume of gas in the right posterior pararenal space adjacent to the stable psoas haematoma, with extension superiorly into the retroperitoneum and inferiorly into the pelvis (Figure 2, Table 1).

**Table 1:** Laboratory trends demonstrating progressive inflammatory response following blunt abdominal trauma. This table demonstrates the patient’s progressive inflammatory response, highlighting delayed clinical deterioration consistent with occult small bowel injury. Admission labs were relatively normal, with a marked rise by day 7, correlating with the development of sepsis and retroperitoneal abscess formation. Labs improved after operative intervention and antimicrobial therapy. CRP, C-reactive protein

Time point	WBC (×10⁹/L)	CRP (mg/L)	Lactate (mmol/L)	Base deficit (mmol/L)
Admission (Day 0)	9.5	12	1.8	-1.0
Interval CT (36 hours post-trauma)	10.2	25	1.9	-1.2
Day 7 (clinical deterioration)	18.4	210	3.2	-5.5
Pre-second-look laparotomy	14.8	180	2.5	-3.8
Discharge	8.6	45	1.5	-0.8

The working diagnosis was a perforation on the mesenteric border of the gastrointestinal tract, accounting for posterior gas tracking into the retroperitoneum rather than the peritoneal cavity. Although a duodenal perforation was considered due to the distribution of gas, there were no CT features of duodenal wall oedema, fat stranding, or mucosal disruption, and the patient remained asymptomatic, tolerating oral intake and passing bowel motions.

An urgent oesophagogastroduodenoscopy (OGD) was performed in theatre, which demonstrated a macroscopically normal duodenum. An exploratory laparotomy was therefore undertaken. Dense intra-abdominal adhesions from previous surgery were encountered. The duodenum was carefully inspected and appeared normal. Mobilisation of a loop of ileum tethered to the pelvic floor revealed a large pelvic abscess cavity tracking superiorly through the retroperitoneum, anterior to the aorta and inferior vena cava. The involved 9 cm ileal segment was bruised and deemed non-viable, although no discrete perforation was identified. This segment was resected. The abscess cavity and abdomen were thoroughly irrigated, and an open abdomen negative-pressure dressing was applied.

A planned second-look laparotomy 36 hours later confirmed healthy, viable bowel, allowing for a side-to-side anastomosis and definitive abdominal closure. The open abdomen was applied due to contamination from the pelvic abscess, borderline bowel viability in the resected segment, and the planned second-look laparotomy to confirm bowel viability before definitive closure.

Histopathology confirmed ischaemic necrosis. Abscess cultures grew Escherichia coli and Enterococcus faecalis. Broad-spectrum intravenous antibiotics were administered and adjusted according to sensitivities.

Postoperatively, the patient recovered uneventfully and was discharged on postoperative day 21. Post-discharge, the patient underwent targeted pelvic fracture rehabilitation, including physiotherapy for mobility and weight-bearing precautions. At the six-week follow-up, the patient demonstrated full recovery without functional limitations (Table 2).

**Table 2:** Timeline of clinical events, investigations and interventions. This timeline demonstrates the evolution of occult small bowel injury and delayed retroperitoneal abscess following blunt abdominal trauma. Serial imaging and laboratory monitoring facilitated timely recognition and intervention, illustrating the dynamic nature of this injury.

Day/Time	Event/Investigation	Findings/Intervention	Clinical relevance
Day 0 (Injury)	Road traffic collision	Pelvic fractures, right lower quadrant tenderness	Initial trauma; patient haemodynamically stable
Day 0	Transfer to MTC	Tachycardia 101 bpm, BP 115/75 mmHg	Patient stable; high-dependency monitoring initiated
Day 2 (36 hours post-trauma)	Interval CT abdomen (IV contrast, portal venous phase)	Retroperitoneal gas in the right posterior pararenal space, mesenteric haematoma adjacent to psoas, no free intraperitoneal air	Early subtle sign of occult bowel injury; no definitive perforation
Day 7	Clinical deterioration	Pyrexia, tachycardia; WBC 18.4 × 10⁹/L, CRP 210 mg/L, lactate 3.2 mmol/L, base deficit -5.5	Suspicion of delayed bowel injury; repeat imaging performed
Day 7	Repeat CT abdomen (IV and oral contrast, portal venous phase)	Interval increase in retroperitoneal gas, new pelvic abscess	Prompted urgent intervention
Day 7	OGD	Normal duodenum	Ruled out duodenal perforation
Day 7	Exploratory laparotomy	9-cm non-viable ileum, mesenteric hematoma, pelvic abscess; abscess cultured	Ileal resection, irrigation, and open abdomen were applied for the planned second-look
Day 9 (36 hours later)	Second-look laparotomy	Healthy bowel; side-to-side anastomosis performed, abdomen closed	Confirmation of bowel viability and definitive closure
Day 21	Discharge	Stable, recovering, outpatient rehab initiated	Full recovery; outpatient follow-up planned

## Discussion

Blunt bowel and mesenteric injuries are uncommon but clinically significant sequelae of blunt abdominal trauma. Although the reported incidence is approximately 1%, delayed diagnosis is associated with substantial morbidity, including sepsis, prolonged hospitalisation and increased mortality [2-4]. This case highlights the diagnostic challenges posed by delayed occult bowel injury and underscores the importance of sustained vigilance, particularly in patients with complex pelvic trauma and prior abdominal surgery.

Contrast-enhanced CT is the cornerstone of evaluation in haemodynamically stable trauma patients. While CT demonstrates high sensitivity for solid organ injury, its sensitivity for hollow viscus injury remains limited, ranging between 55% and 80% in large series [5,6]. Classical CT findings such as free intraperitoneal air, bowel wall discontinuity, or contrast extravasation are infrequently observed in the acute setting. Instead, clinicians must often rely on indirect and non-specific signs, including mesenteric fat stranding, bowel wall thickening, isolated free fluid or mesenteric haematoma [6-8].

In the present case, early CT findings were confounded by the presence of a psoas haematoma and extensive pelvic fractures, which may have obscured subtle mesenteric injury. Additionally, the retroperitoneal distribution of gas rather than intraperitoneal free air further reduced the diagnostic specificity of CT imaging.

Delayed bowel injury following blunt trauma has been attributed to several pathophysiological mechanisms. These include mural contusion with progressive transmural necrosis, mesenteric vascular injury leading to delayed ischaemia, and partial-thickness bowel tears that subsequently progress to perforation [9,10]. These mechanisms may explain the absence of an identifiable macroscopic perforation in this patient, despite the presence of non-viable bowel and abscess formation.

Several studies have described delayed bowel necrosis and abscess formation without visible perforation, suggesting that bacterial translocation across a compromised bowel wall or microperforation may play a significant role in the development of sepsis [10,11]. This phenomenon likely accounts for the progressive retroperitoneal gas and pelvic abscess observed in this case.

Retroperitoneal gas following blunt trauma is an uncommon but important radiological finding. Although it is classically associated with duodenal injury, posterior injuries to the small bowel or colon and infected retroperitoneal haematomas must also be considered [12]. In the absence of supporting CT features such as duodenal wall thickening, periduodenal fluid, or contrast leak, the diagnostic specificity of retroperitoneal gas is limited [13].

In this case, the retroperitoneal gas distribution initially raised suspicion for duodenal injury. However, the absence of corroborative radiological and endoscopic findings, combined with the intra-operative discovery of a pelvic abscess tracking retroperitoneally, suggests a posterior small bowel injury as the most likely source.

Previous abdominal surgery complicates the assessment of trauma patients by altering normal anatomical planes and promoting adhesion formation. These changes can obscure clinical signs, alter expected imaging findings, and facilitate atypical spread of gas or infection [14]. In this patient, prior colorectal surgery likely contributed to abnormal bowel fixation within the pelvis and atypical retroperitoneal tracking of gas, delaying diagnosis.

The diagnosis of blunt bowel injury is often dynamic rather than static. Multiple studies emphasise the importance of serial clinical examinations, laboratory monitoring, and repeat imaging when initial findings are equivocal [3,4,15]. Clinical deterioration - manifested by pyrexia, tachycardia or rising inflammatory markers - should prompt reconsideration of operative intervention, even in the absence of overt peritonitis. In this case, serial laboratory trends facilitated recognition of delayed sepsis.

Diagnostic laparoscopy is a useful adjunct in selected patients with equivocal findings; however, extensive prior abdominal surgery may limit its feasibility and diagnostic yield [16]. In such cases, early exploratory laparotomy remains the definitive diagnostic and therapeutic approach once clinical deterioration occurs. Operative findings confirmed ischaemic ileum without an identifiable perforation, highlighting the role of transmural necrosis and bacterial translocation.

This case reinforces the need for a low threshold for re-imaging and surgical exploration in patients with unexplained radiological findings following blunt trauma. Patients with pelvic fractures and retroperitoneal haematomas are at increased risk of associated bowel and mesenteric injury and warrant close observation [17,18]. This case also underscores the importance of careful interpretation of subtle radiologic findings (retroperitoneal gas and mesenteric haematoma) and serial clinical assessment and laboratory monitoring.

## Conclusions

Delayed bowel injury following blunt abdominal trauma can evolve despite initially reassuring imaging and clinical stability. This case demonstrates that significant gastrointestinal pathology can evolve despite initial clinical stability and reassuring imaging. Progressive retroperitoneal gas and rising inflammatory markers may precede sepsis, emphasising the need for repeated assessment and timely intervention. Clinicians should maintain a low threshold for re-imaging and operative exploration in patients with atypical presentations or prior abdominal surgery. Observations from this case are hypothesis-generating and highlight clinical vigilance rather than definitive predictive rules.
